# A *Gossypium hirsutum* GDSL lipase/hydrolase gene (*GhGLIP*) appears to be involved in promoting seed growth in Arabidopsis

**DOI:** 10.1371/journal.pone.0195556

**Published:** 2018-04-05

**Authors:** Rendi Ma, Hali Yuan, Jing An, Xiaoyun Hao, Hongbin Li

**Affiliations:** 1 College of Life Sciences, Key Laboratory of Agrobiotechnolog, Shihezi University, Shihezi, Xinjiang, China; 2 Key Laboratory of Xinjiang Phytomedicine Resource Utilization, Ministry of Education, Shihezi University, Shihezi, Xinjiang, China; Institute of Genetics and Developmental Biology Chinese Academy of Sciences, CHINA

## Abstract

GDSL lipase (GLIP) plays a pivotal role in plant cell growth as a multifunctional hydrolytic enzyme. Herein, a cotton (*Gossypium hirsutum* L. cv Xuzhou 142) GDSL lipase gene (*GhGLIP*) was obtained from developing ovules and fibers. The *GhGLIP* cDNA contained an open reading frame (ORF) of 1,143 base pairs (bp) and encodes a putative polypeptide of 380 amino acid residues. Sequence alignment indicated that GhGLIP includes four enzyme catalytic amino acid residue sites of Ser (S), Gly (G), Asn (N) and His (H), located in four conserved blocks. Phylogenetic tree analysis showed that GhGLIP belongs to the typical class IV lipase family with potential functions in plant secondary metabolism. Subcellular distribution analysis demonstrated that GhGLIP localized to the nucleus, cytoplasm and plasma membrane. *GhGLIP* was expressed predominantly at 5–15 day post anthesis (dpa) in developing ovules and elongating fibers, measured as mRNA levels and enzyme activity. Ectopic overexpression of *GhGLIP* in Arabidopsis plants resulted in enhanced seed development, including length and fresh weight. Meanwhile, there was increased soluble sugar and protein storage in transgenic Arabidopsis plants, coupled with the promotion of lipase activity. Moreover, the expression of cotton *GhGLIP* is induced by ethylene (ETH) treatment *in vitro*. A 1,954-bp *GhGLIP* promoter was isolated and expressed high activity in driving green fluorescence protein (GFP) expression in tobacco leaves. *Cis*-acting element analysis of the *GhGLIP* promoter (*pGhGLIP*) indicated the presence of an ethylene-responsive element (ERE), and transgenic tobacco leaves with ectopic expression of *pGhGLIP*::*GFP-GUS* showed increased GUS activity after ETH treatment. In summary, these results suggest that GhGLIP is a functional enzyme involved in ovule and fiber development and performs significant roles in seed development.

## Introduction

Upland cotton (*Gossypium hirsutum* L.*)* is a vital economic crop worldwide, and cotton seeds and fibers are among the most important materials for the oil and textile industry. The fiber, a single-celled attachment developed from ovule epidermal cells, is inseparable with ovule growth [1–2]. Many factors affect ovule and fiber development, including sugar biosynthesis, lipid metabolism, the plant hormone ethylene, etc. Sugar is essential for ovule and fiber growth; upregulating or downregulating sucrose synthase (*SuSy*) expression results in increased or reduced sugar content of the cell, which promotes or represses ovule development and fiber elongation [3–5]. Several fatty acid biosynthesis-related genes promote ovule and fiber development by enhancing cellular fatty acid content [6–9]. Ethylene facilitates fiber elongation by inducing *SuSy* expression and responding to the signal of very long chain fatty acid (VLCFA) [6, 10].

GDSL lipases are important subclasses or subfamilies of the lipolytic enzymes and exist widely in microorganisms [11]. In plants, GDSL lipases are found as multifunctional enzymes and participate in many physiological processes including plant growth, morphogenesis and pathogen response [12–16]. The first plant GDSL lipase gene was isolated from *Brassica napus* [17]. A total of 105, 121 and 114 GDSL esterase/lipase genes have been reported in Arabidopsis, *Brassica napa* and rice (*Oryza sativa* L. japonica), respectively [18–20]. Arabidopsis GDSL lipase genes *GLIP1* and *GLIP2* were found to be important for regulating systemic resistance and pathogen defense, respectively [16, 21–22]. Rice *GER1*, a GDSL-motif encoding gene, regulates coleoptile elongation by light-JA interaction. A member of the GDSL lipase family, WDL1, has a substantial role in rice wax and epidermal cell layer biosynthesis [23]. Pepper GDSL lipase performs a significant function in responding to abiotic stresses and pathogen defense [24–25]. Tomato GDSL1 protein is essential for extracellular deposition of the cutin polyester in fruit cuticle [26].

Studies have reported that GDSL lipase has crucial roles in seed development. Two GDSL lipase genes *BnLIP1* and *BnLIP2* of *Brassica napus* may exhibit pivotal roles in rapeseed germination and morphogenesis [17]. The Arabidopsis *RGE1* gene is expressed in endosperm and cooperates with the GDSL lipase gene to control embryo growth and seed morphology [27]. Arabidopsis *EXL4* promotes hydration on the stigma surface as well as ovule fertilization by changing lipid composition, which affects pollen tube growth and controls the seed growth [13]. The induction of expression and function of GDSL lipase in response to ethylene has been observed [21].

In higher plants, GDSL lipase is a novel member of the lipase superfamily, and its diverse functions and molecular mechanisms are still unclear. Promoter fragment of the *Gossypium hirsutum* GDSL (*GhGDSL*) lipase/hydrolase gene showed stage-specific high activity and was regulated by transcription factor GhMYB1 to involve in secondary cell wall biosynthesis of fiber development [28]. However, further functional analysis of cotton GDSL lipase gene is rarely reported. In this study, we obtained a GDSL lipase gene *GhGLIP* that was expressed predominantly in developing ovules and elongating fibers, with the encoded protein localizing in the nucleus, cytoplasm and plasma membrane. Overexpression of *GhGLIP* in Arabidopsis resulted in the enhancement of seed length and weight, following the accumulation of soluble sugar and protein content. The *GhGLIP* expression was significantly induced after ETH treatment; meanwhile, the functional 1,954-bp *GhGLIP* promoter containing one ERE element indicated high activity driving green fluorescence protein (GFP) expression, with a significant GUS activity increase in response to ethylene stimulation. We conclude that cotton *GhGLIP* may be involved in ovule and fiber development and promote seed growth.

## Materials and methods

### Plant material and growth condition

Upland cotton (*Gossypium hirsutum* L. cv Xuzhou 142) wild type (WT) and *fuzzless-lintless* (*fl*) mutant were planted in the experimental field of Shihezi university with the location in the northwest of shihezi city (44°19'46"N, 85°59'50"E). Blooming flowers were labeled on the day of anthesis, and samples of WT cotton ovules and fibers at -3, 0, 3, 5, 10, 15, 20, 30 days post anthesis (dpa) and of 15 dpa *fl* mutant ovules, as well as roots, stems and leaves, were collected and frozen in liquid nitrogen for preservation and further use.

The seeds of *Arabidopsis thaliana* (ecotype Columbia-0, Col-0) were plated on half-strength Murashige and Skoog solid medium after sterilizing with 75% ethanol and 10% sodium hypochlorite. Approximately 10 d later, Arabidopsis plants were transplanted in nutritious soil and cultivated in artificial climatic incubators under controlled conditions (16 h light/8 h darkness cycle at 20 2°C) for normal growth.

The tobacco (*Nicotiana benthamiana*) seeds were sterilized with 75% ethanol and 10% sodium hypochlorite and then plated on MS solid medium in a petri dish. After 2–3 days of vernalization at 4°C, the petri dishes were placed in artificial climate incubators at 28°C with a 16 h light/8 h darkness cycle. The tobacco plants were transferred to incubation bottles containing solid MS medium when 2–3 pieces of true leaves appeared. After approximately 15 days growth, the tobacco plants were transplanted to nutrient soil and taken for instantaneous conversion using leaves as materials.

### Sequence analysis

Protein sequences were obtained from the GenBank database maintained by the National Center for Biotechnology Information (NCBI) and aligned using the ClustalX program version 2.1 (European Bioinformatics Institute, Cambridge, UK) with default settings. The online software of conserved domains (CD) on the NCBI website (https://www.ncbi.nlm.nih.gov/Structure/cdd/wrpsb.cgi), and SignalP and TMpred on the ExPASy website (http://www.expasy.org/) were used for prediction analyses of conserved domains, signal peptides and transmembrane helixes, respectively.

### Promoter *cis*-element analysis

Through searching the cotton genome database at Phytozome (https://phytozome.jgi.doe.gov/pz/portal.html) using the *GhGLIP* coding sequence (GenBank accession number MG437049) as the alignment reference, a 1,954-bp promoter sequence upstream to the *GhGLIP* translational start codon was isolated. The promoter region of *GhGLIP* (*pGhGLIP*) was analyzed by PlantCARE (http://bioinformatics.psb.ugent.be/webtools/plantcare/html/) to obtain the *cis*-acting regulatory elements.

### Vector construction

To construct the overexpression vector *35S*::*GhGLIP-GFP*, the *GhGLIP* cDNA, amplified using reverse transcription polymerase chain reaction (RT-PCR) with specific primers (listed in S1 Table) carrying *Kpn*I and *Xba*I restriction endonuclease sites, was integrated upstream to the green fluorescent protein (GFP) gene in the modified expression vector pCAMBIA2300-GFP after double enzymatic digestion and ligation. The validated vector *35S*::*GhGLIP-GFP* was used for Arabidopsis genetic transformation and subcellular localization analysis.

The *pGhGLIP* and the modified vector pCAMBIA1304-GFP-GUS were utilized to construct the recombinant vector *pGhGLIP*::*GFP-GUS*. We designed specific primers (listed in S1 Table) containing the restriction endonulcease sites *Bam*HI and *Nco*I to enable the insertion of *pGhGLIP* into the modified vector pCAMBIA1304-GFP-GUS, replacing the cauliflower mosaic virus (CaMV) 35S promoter. The newly generated *pGhGLIP*::*GFP-GUS* vector allowed successive GFP detection and GUS activity analysis in transgenic tobacco leaves through the Agrobacterium-mediated transient transformation method.

### GFP localization analysis

The constructed vector *35S*::*GhGLIP-GFP* was transformed into onion epidermal cells to analyze *GhGLIP* subcellular distribution based on a previously reported method [29]. After treatment with 75% ethanol and sterilized water, the epidermal cells from onion leaves were isolated and transformed with *35S*::*GhGLIP-GFP* through an Agrobacterium-mediated electroporation method. The transformed onion epidermal cells were subcultured for 24 h and then analyzed for GFP signal with confocal laser-scanning microscope (Zeiss LSM 510, Oberkochen, Germany).

The tobacco leaves transformed with *Agrobacterium tumefaciens* (strain GV3101) containing the *pGhGLIP*::*GFP-GUS* vector through the transient transformation method were utilized for GFP detection. The GFP fluorescence signal was observed under laser excitation with confocal laser-scanning microscope (Zeiss LSM 510, Oberkochen, Germany) at a wavelength of 488 nm.

### qRT-PCR analysis

Total RNA was extracted from various materials including fibers and ovules at different developmental stages, roots, stems and leaves, and then was used for cDNA synthesis through reverse transcription. The cDNA was used as template for PCR analysis. qRT-PCR was performed on the Light Cycler 480 (Roche, Rotkreuz, Switzerland) using the SYBR^®^Premix Ex TaqTM kit (TaKaRa, Kusatsu, Japan) with specific primers (listed in S1 Table) and using the cotton ubiquitin 7 (*GhUBQ7*) gene as internal control.

### Transformation of tobacco and Arabidopsis plants

The constructed vectors of *35S*::*GhGLIP-GFP* and *pGhGLIP*::*GFP-GUS* were introduced into *Agrobacterium tumefaciens* (strain GV3101) by electroporation and were then used to transform *Arabidopsis thaliana* and tobacco (*Nicotiana benthamiana*) leaves, respectively. The transformation of *Arabidopsis thaliana* was performed with a floral dip method [30]. After kanamycin screening to obtain resistant plants and selfing to acquire the homozygous plant lines, the generated T3 generation transgenic Arabidopsis overexpression lines (OEs) with *GhGLIP* stable and high expression level detected by RT-PCR were used for further analysis.

The transformation of tobacco leaves was carried out with the Agrobacterium-mediated transient transformation method [29]. After the transformation was completed, the transgenic tobacco plants were placed under normal light conditions for 3–5 days, and then the leaves were collected for GFP detection and GUS enzyme activity analysis.

### Protein extraction and enzyme activity assay

Approximately 0.2 g of various materials of cotton plant and transgenic tobacco leaves were ground to powder in liquid nitrogen and then were transferred to a centrifuge tube containing 2 mL pre-cooled enzyme extract mix (50 mM phosphate buffer, pH 7.0). After centrifugation at 12,000 rpm for 15 min at 4°C, the supernatant was collected for determination of the protein content and enzyme activities of lipase and GUS. The Bradford method was used to quantify the protein concentration using bovine serum albumin (BSA) as a standard protein, using an ultraviolet spectrophotometer to measure the absorbance at 280 nm [31].

Approximately 50 g protein extract was added into a reaction system including 50 mM sodium phosphate buffer (pH 7.0), 1 mM P-nitrophenol laurate and 5% (v/v) isopropanol. After an adequate reaction time of 15 min at 37C, the reaction mix was heated at 100°C for 1–2 min and placed at room temperature for natural cooling. Then, the reaction mix was used for lipase activity determination by measuring the change in absorbance at 405 nm by ultraviolet spectrophotometer. The lipase activity unit was defined as 1 mmol p-nitrophenol per mg protein per min.

GUS activity was measured as previously described [32]. The protein extract was mixed with GUS assay buffer containing 2 mM 4-methylumbelliferyl-b-glucuronide (4-MUG), 50 mM sodium phosphate buffer pH 7.0, 10 mM β-mercaptoethanol, 10 mM Na_2_EDTA, 0.1% sodium lauroyl sarcosine, and 0.1% Triton X-100. The assay was performed at 37°C until completion of the reaction and quenched by adding 0.2 M Na_2_CO_3_. GUS activity was determined by monitoring the generation of 4-methylumbelliferone fluorochrome (4-MU) using a fluorescence spectrophotometer at an excitation wavelength at 365 nm and an emission wavelength at 455 nm. The GUS activity was defined as nmol of 4-MU per mg protein per min.

### Soluble sugar determination

Soluble sugar content was measured by the Anthrone method [33]. Arabidopsis seeds were collected and ground to powder in liquid nitrogen, and approximately 0.5 g of material was placed in a 25 mL tube with 10 mL of distilled water. The mixture was incubated at 100°C for 1 h followed by filtration into a conical flask. 0.5 mL extract was added into the reaction mixture including 0.5 mL mixed solution (1 g anthrone and 50 mL ethylacetate), 5 mL 98% H_2_SO_4_, and 1.5 mL distilled water. The reaction mixture was heated at 100°C for 1 min and the absorbance was measured at 630 nm by ultraviolet spectrophotometer, with the sucrose as a standard sample.

### Ethylene treatment

*In vitro* ovule culture and ETH treatment were performed as described [34]. Cotton fertilized ovules at 1 DPA were peeled from the boll and then washed with 70% ethanol, distilled and deionized water, and 0.1% HgCl_2_ solution containing 0.05% Tween-80, successively. Approximately 20 sterilized ovules were put in a 100 mL conical flask containing 20 mL liquid culture media at 30°C under darkness without agitation, and the liquid media was supplemented with or without 0.1 M ethylene precursor 1-aminocyclopropane-1-carboxylic acid (ACC). The ovules were independently treated in triplicate and were used for total RNA extraction and further qRT-PCR analysis.

The transgenic tobacco plants transformed with *pGhGLIP*::*GFP-GUS* using the transient conversion method were grown in an artificial climate incubator. The leaves of 6- to 7-week-old tobacco plants were treated with white fluorescent lamp for 1 h followed by 1 M ethylene for 2 days, generating the treated materials for GUS activity analysis.

## Results

### Identification of cotton GDSL lipase

**Fig 1:**
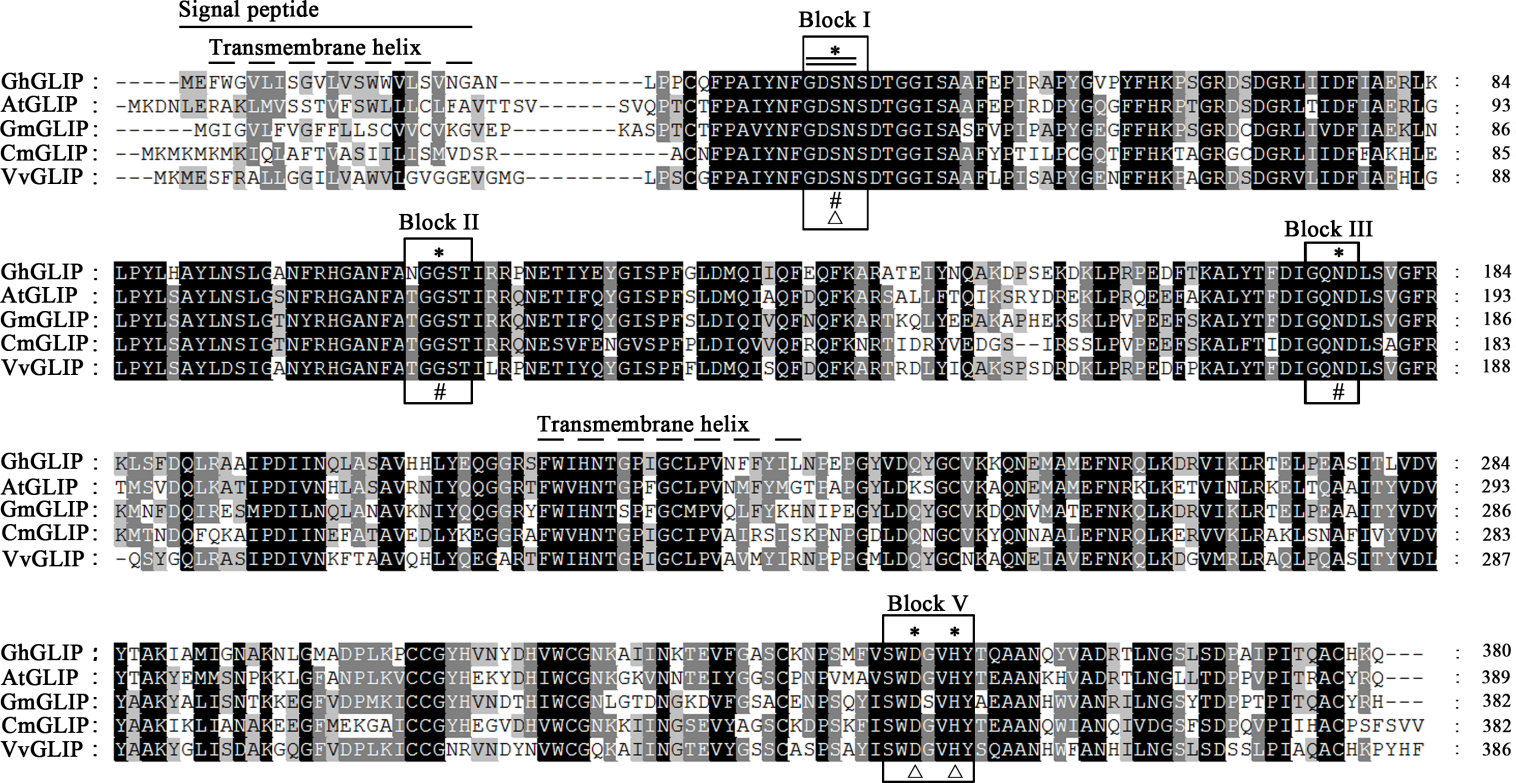
Sequence alignment and conserved domain analysis of GhGLIP. Four conserved blocks of I, II, III and V were boxed. The typical GDS(L) sequence is indicated with a double line. The N-terminal signal peptide and transmembrane helix are displayed with solid and dashed lines respectively. The symbols asterisks (*), triangles () and pounds (#) represent the plant SGNH-lipase conserved active sites residues, Ser-His-Asp(Glu) catalytic triad and oxyanion hole respectively. The aligned proteins are listed in S2 Table.

Cotton *GhGLIP* cDNA, containing a 1,143-bp open reading frame (ORF) and encoding a putative protein of 380 amino acid residues with a theoretical molecular (MW) weight of 41.8 kDa, was cloned from developing ovules and fibers using RT-PCR. As shown in Fig 1, in alignment with homologous sequences from species including *Arabidopsis thaliana*, *Glycine max*, *Cucumis melo* and *Vitis vinifera*, the GhGLIP included conserved functional domains and belonged to a plant specific subfamily of the SGNH_hydrolase (GDSL_hydrolase) superfamily that is known as a diverse family of lipases and esterases.

**Fig 2:**
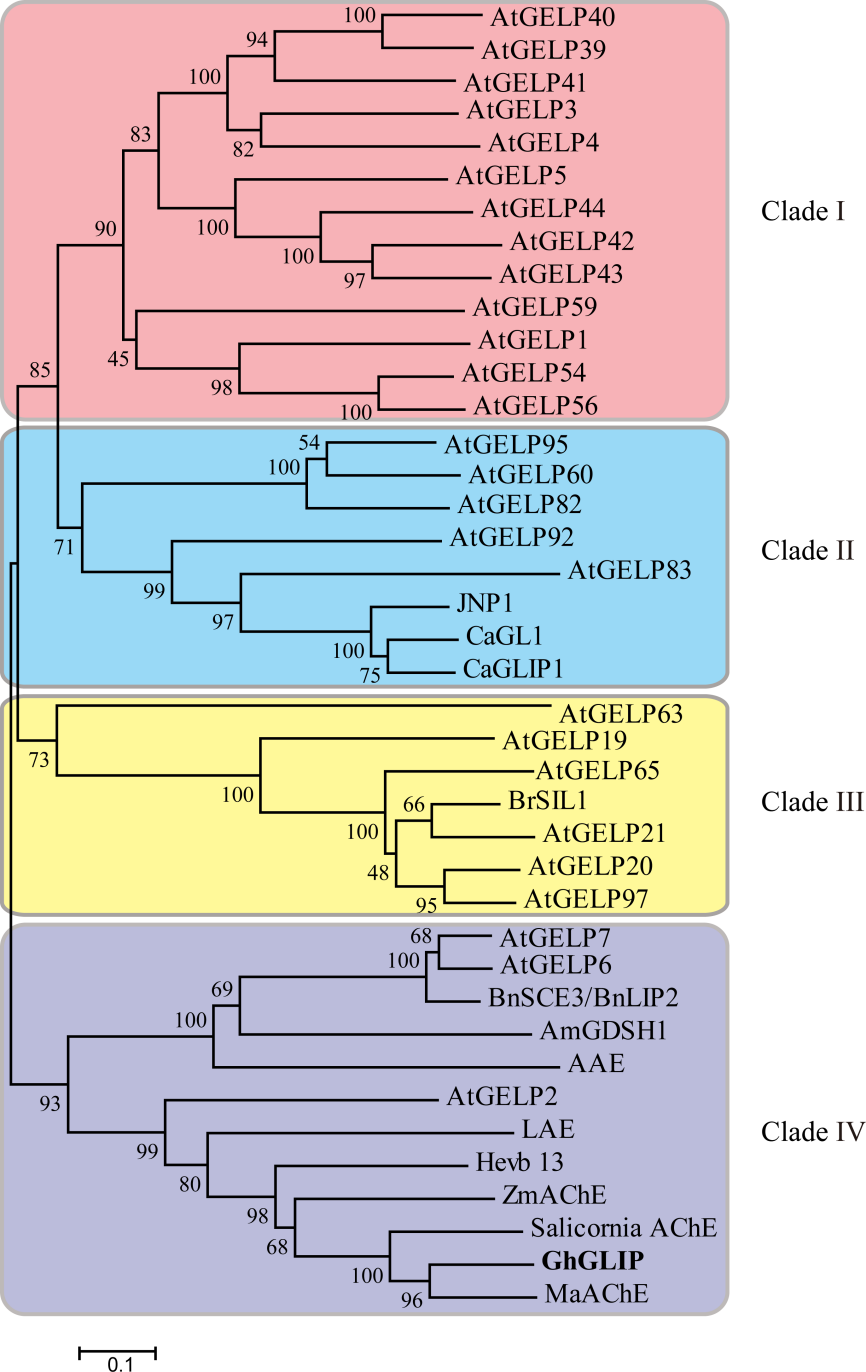
Phylogenetic relationship of GDSL lipase in plants. The unrooted phylogenetic tree was constructed with the ClustalW and MEGA 5.0 programs using the NJ method, with 1,000 bootstrap replicates. Classified clades are shown at the right part of the tree and marked with different tones of background. GhGLIP is highlighted in bold. The proteins are selected from the Arabidopsis GLIP family and other identified plant GDSL lipases, with GenBank accession numbers listed in S2 Table.

The GhGLIP protein possessed a signal peptide at the N terminus containing amino acid residues 1–22 and two transmembrane helixes containing amino acid resides 3–30 and 217–236, implying that GhGLIP is a secretory protein. GhGLIP included the 4 functional amino acid residues Ser(S), Gly(G), Asn(N) and His(H) distributed in 4 conserved regions of block I, II, III and V respectively, which perform pivotal functions in catalyzing enzyme reaction through formation of a Ser-His-Asp(Glu) triad. In addition, 5 active sites (S, G, D, N and H), 1 catalytic triad composed of Ser-His-Asp(Glu) and 4 oxyanion holes (S, G, N and H) were indicated (Fig 1). These data provide the possibility that GhGLIP may function in hydrolase/esterase reactions as a transmembrane protein. Based on the homologous plant GLIP protein sequences, a phylogenetic tree constructed using the neighbor-joining (NJ) method showed that the GLIP proteins can be divided into four groups, and GhGLIP belongs to clade IV (Fig 2) with putative roles in plant secondary metabolism.

### *GhGLIP* is predominantly expressed during ovule and fiber development

**Fig 3:**
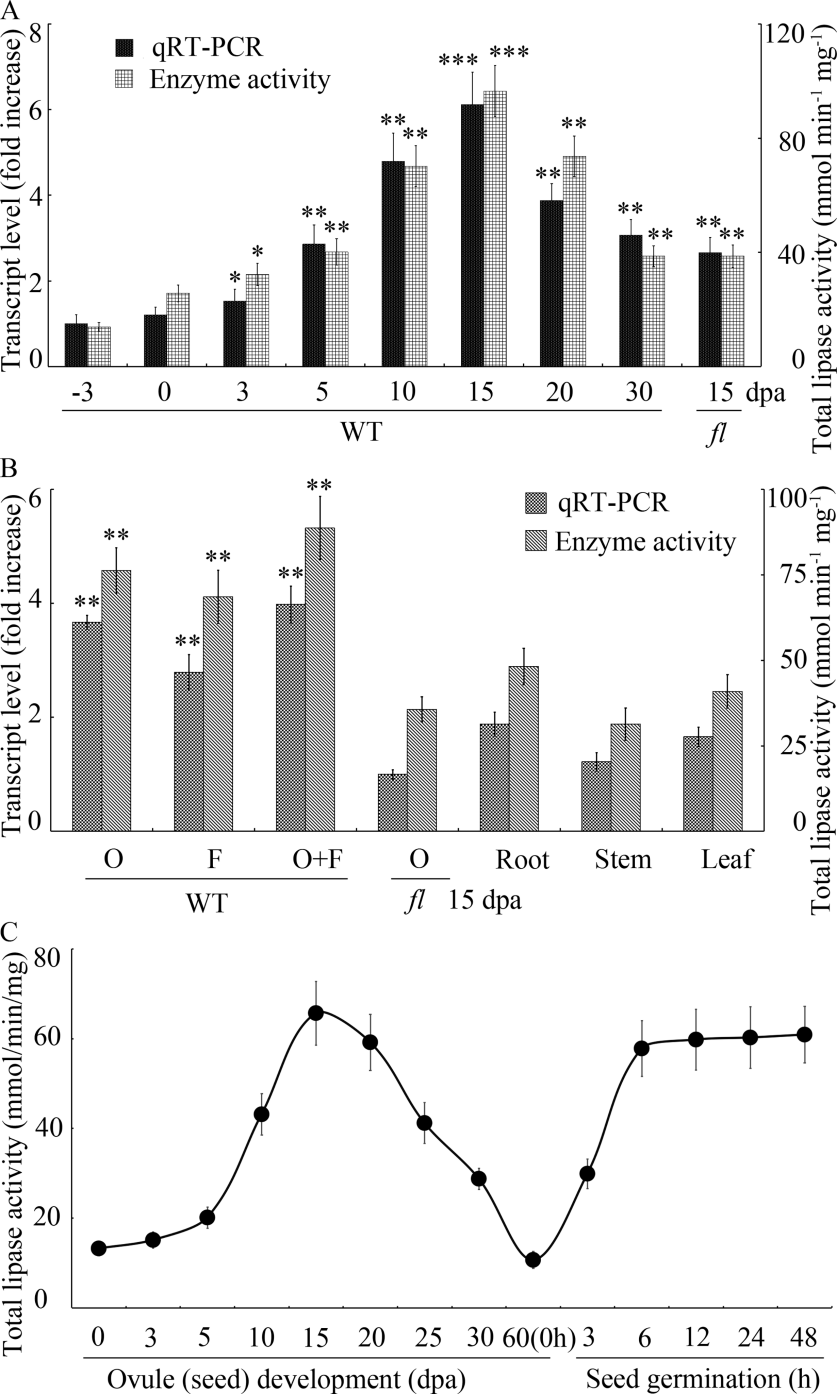
Expression pattern analysis of *GhGLIP* during cotton ovule and fiber development. (A) *GhGLIP* is preferentially expressed in ovule enlargement and fiber elongating stages. qRT-PCR and analysis of lipase enzyme activity were carried out using total RNA and protein extracted from materials of wild-type cotton ovules associated with fibers at -3,0, 3, 5, 10, 15, 20, 30 dpa and 15 dpa fl mutant ovules. qRT-PCR data from -3 dpa WT ovules were artificially set to 1. (B) Tissue-specific analysis of *GhGLIP* in different cotton materials. Various tissues of cotton plants containing ovules (O), fibers (F), and ovules associated with fibers (O+F) of 15 dpa WT, and 15 dpa fl mutant ovules, as well as roots, stems, and leaves, were used for measurement of qRT-PCR and enzyme activity. qRT-PCR data from 15 dpa *fl* mutant ovules were artificially set to 1. (C) Determination of lipase activity of cotton ovules stripped fibers during ovule maturation and seed germination stages. Lipase activity was detected by monitoring the absorbance at 405 nm using protein samples extracted from different cotton tissues presented. The cotton ubiquitin gene *GhUBQ7* (GenBank accession no. AY189972) was used as endogenous reference gene for qRT-PCR analysis. Error bars display the standard error from three independent experiments. Data of qRT-PCR and lipase activity from -3 dpa WT ovules (in A) and 15 dpa *fl* mutant ovules (in B) were used as references for analysis of statistical difference test. Asterisks indicate significant differences according to Student's *t* tests, *p<0.05; **p<0.01; ***p<0.001.

To understand the important functions of *GhGLIP* in cotton ovule and fiber development, qRT-PCR and enzyme activity were used to probe *GhGLIP* expression patterns in different ovule and fiber developmental stages. In comparison with -3 dpa ovules, *GhGLIP* showed over a 6-fold increase in 15 dpa (the fastest point of ovule expanding and fiber elongation) WT ovules associated with fibers at mRNA level. The total lipase enzyme activity reached the peak value at 15 dpa, consistent with the transcriptional expression level of the gene (Fig 3A). A tissue-specific pattern was analyzed in roots, leaves, stems and 15-dpa ovule-associated fibers. qRT-PCR results using RNA extracts from the different samples indicated that *GhGLIP* was mainly expressed in WT ovule and fiber tissues with relatively low mRNA levels in other tissues. The total lipase enzyme activity demonstrated similar expression patterns as qRT-PCR data (Fig 3B). To study *GhGLIP* expression features involved in seed (matured ovule) growth in-depth, developing seeds isolated from different stages of ovule development and seed germination were analyzed for lipase activity. During seed development, lipase activity achieved the peak value at 15 dpa and then declined gradually, and then showed a prompt increase at the seed germination stage (Fig 3C), which suggests GhGLIP potential important role in seed development and germination. These results demonstrate that GhGLIP accumulates significantly in the process of cotton ovule enlargement and fiber elongation.

### GhGLIP localizes in the plasma membrane, cytoplasm and nucleus

**Fig 4:**
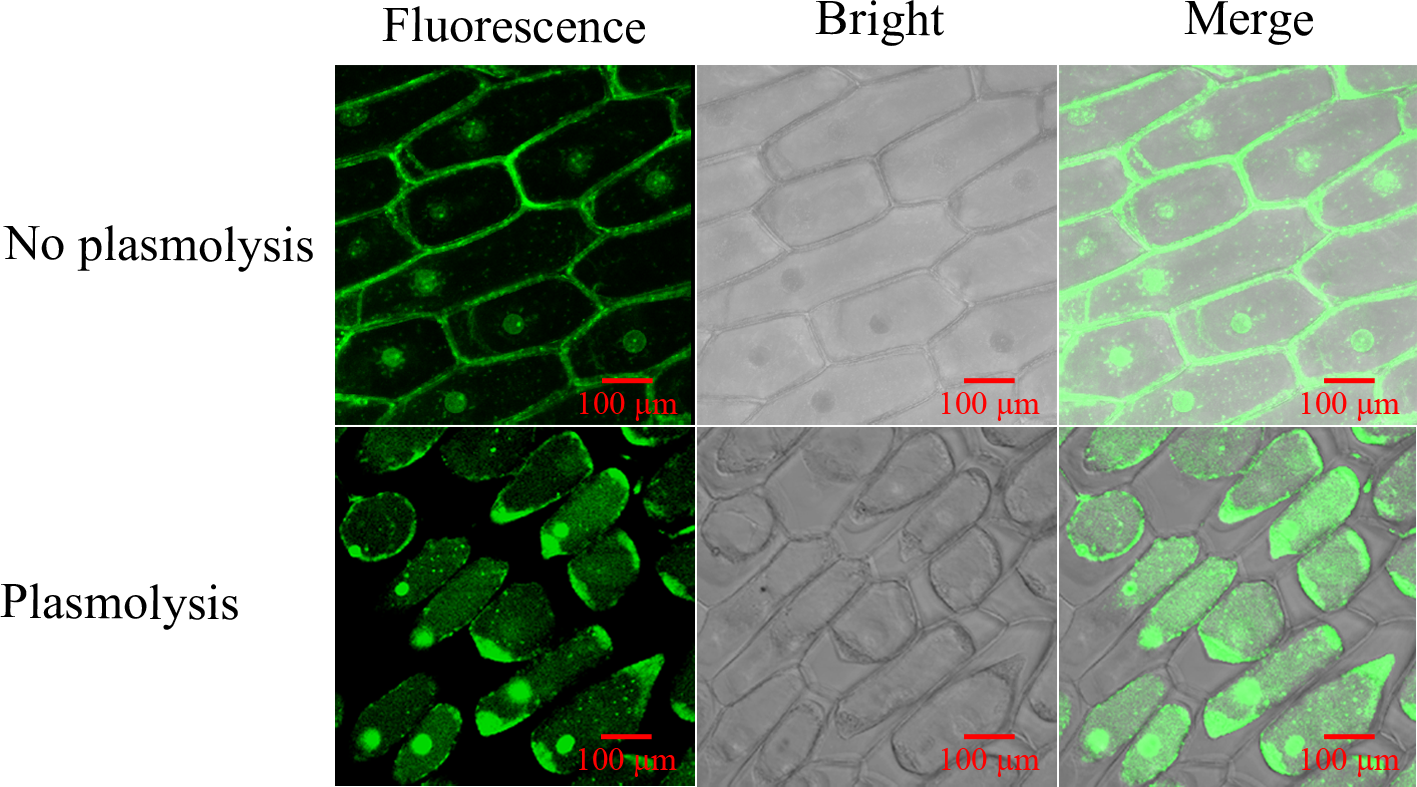
GhGLIP subcellular localization in onion cells. The transformed onion cells were obtained after introduction of *35S*::*GhGLIP-GFP* through the Agrobacterium-mediated method. Plasmolysis was generated after mannitol treatment. Images of different conditions of bright field, fluorescence, and merge were photographed by confocal microscopy.

To examine the subcellular distribution of GhGLIP, the *GhGLIP* gene was cloned into the modified pCAMBIA 2300-GFP vector to generate the *35S*::*GhGLIP-GFP* construct. Ectopic expression of *35S*::*GhGLIP-GFP* was detected after transformation into onion epidermal cells through the Agrobacterium-mediated electroporation method. Fluorescence microscopy showed GFP fluorescence of *35S*::*GhGLIP-GFP* in the extracellular apparatus (plasma membrane or cell wall) and nucleus. Further plasmolysis experiments were used for forward confirmation of GhGLIP localization after mannitol treatment, which indicated that GFP signal was detected in the plasma membrane, cytoplasm and nucleus (Fig 4). The results support the possibility that GhGLIP may function through controlling signal transduction from the outside to the inside of the cell.

### Overexpression of *GhGLIP* promotes Arabidopsis seed growth

**Fig 5:**
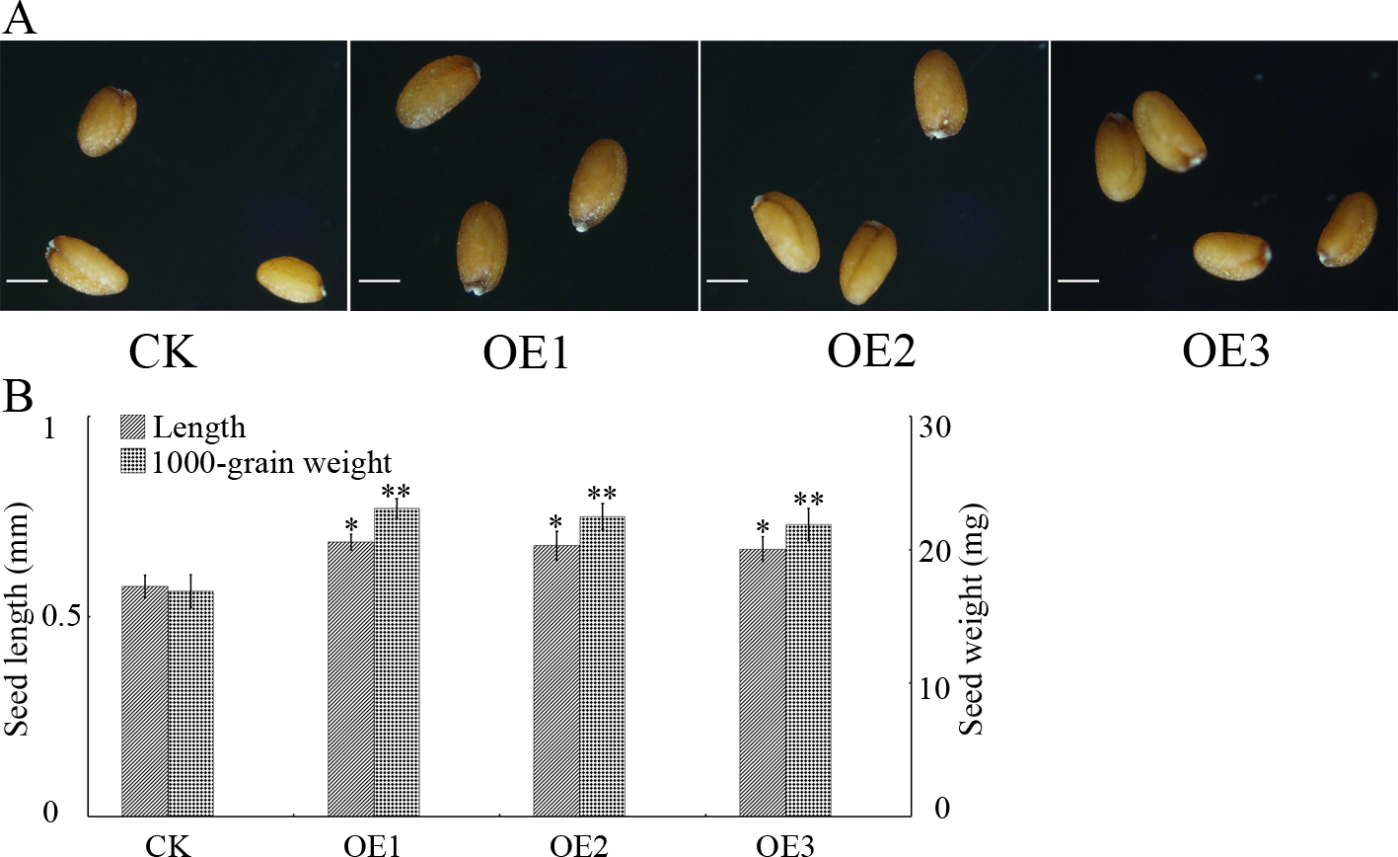
Anaiysis of seed morphology and fresh weight in Arabidopsis *35S*::*GhGLIP* overexpression lines (OEs). (A) Representative seed phenotypes of transgenic Arabidopsis plants with ectopic overexpression of the vector and *35S*::*GhGLIP*. Bar = 0.3 mm. (B) Statistics of seed length and fresh weight. The values are meansSD of 100 seeds selected randomly in three independent experiments. * and ** indicate significant differences according to Student’s *t* tests between CK and OEs at the *p*<0.05 and *p*<0.01 level, respectively.

To investigate the function of *GhGLIP* in plant cell growth, the constructed *35S*::*GhGLIP-GFP* vector was transformed into *Arabidopsis thaliana* (Columbia-0 ecotype) through the floral-dip method. The generated T3 overexpression lines (OEs) were used for further analysis. The transgenic Arabidopsis plants overexpressing *GhGLIP* showed a promotion of plant growth and development (S1 Fig). Concerning the specific expression of *GhGLIP* in ovule development (Fig 3B and 3C), morphology and seed weight were chosen for measurement in the OEs. Among the OEs, seed length was significantly enhanced, with a highest increase ratio of 15.6% (in OE1) while nearly no change of seed width was observed. Meanwhile, seed fresh weight improved with a topmost increase ratio of 35.9% (in OE1) (Fig 5). The results demonstrate that *GhGLIP* plays a positive role in the promotion of plant seed growth.

### *GhGLIP* induces accumulation of protein and soluble sugar

**Fig 6:**
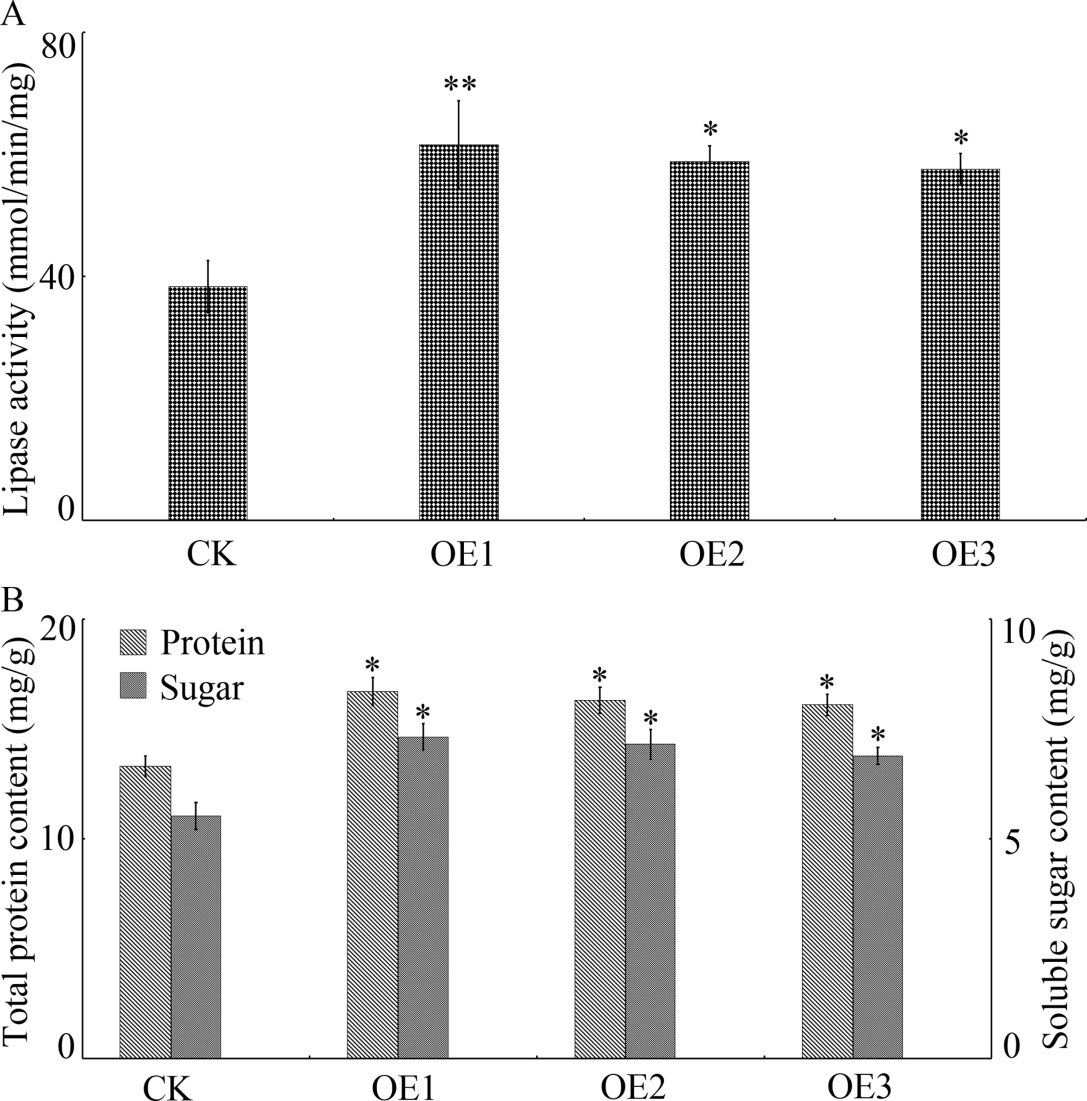
Changes in the levels of lipase activity, soluble sugar and protein content in OEs of Arabidopsis plants. (A) Changes of lipase activity in CK and Arabidopsis OEs. (B) Levels of soluble sugar and protein contents in CK and Arabidopsis OEs. CK, transgenic Arabidopsis plant lines overexpressing the modified pCAMBIA2300-GFP vector; OE, transgenic Arabidopsis plant lines overexpressing *35S*::*GhGLIP*. The mature seeds of Arabidopsis plants were used as samples to determine lipase activity and contents of soluble sugar and protein. Values indicate the average of three independent experiments. Asterisks indicate significant differences according to Student’s *t* tests between CK and OEs, *p<0.05; **p<0.01.

In comparison with controls, lipase activity predominantly showed enrichment in OEs (Fig 6A). Meanwhile, in view of the key function of protein and soluble sugar in seed development [35], we analyzed the change in protein and soluble sugar contents. In transgenic Arabidopsis plants overexpressing *GhGLIP*, both total protein storage and soluble sugar content were significantly higher than in controls with an increase of 14% and 15.81%, respectively (Fig 6B), indicating that *GhGLIP* has an important role in the generation of protein and soluble sugar of the cell, and demonstrating that accumulation of protein and soluble sugar have a substantial role in the enhancement of seed growth.

### *GhGLIP* expression is modulated by ethylene

**Fig 7:**
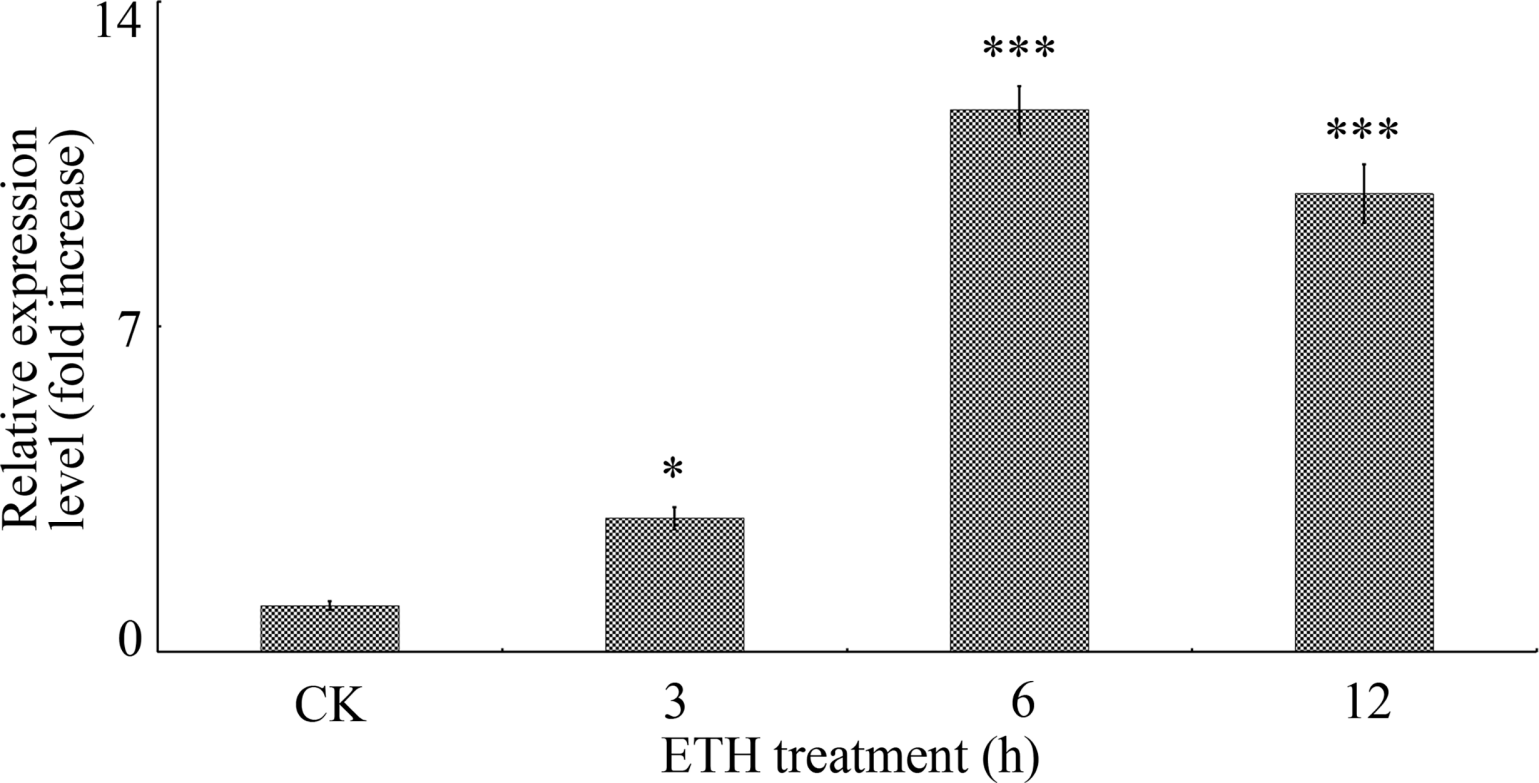
Expression analysis of *GhGLIP* under ethylene treatment. Ovule and fiber tissues treated with 0.1 M ETH through *in vitro* ovule culture were used for RNA extraction and qRT-PCR. The cotton ubiquitin gene *GhUBQ7* was used as endogenous reference gene for qRT-PCR analysis. Error bars display the standard error from three independent experiments. qRT-PCR data of CK were artificially set to 1 and used as reference for analysis of statistical difference test. Asterisks indicate significant differences according to Student's *t* tests, *p<0.05; ***p<0.001.

**Fig 8:**
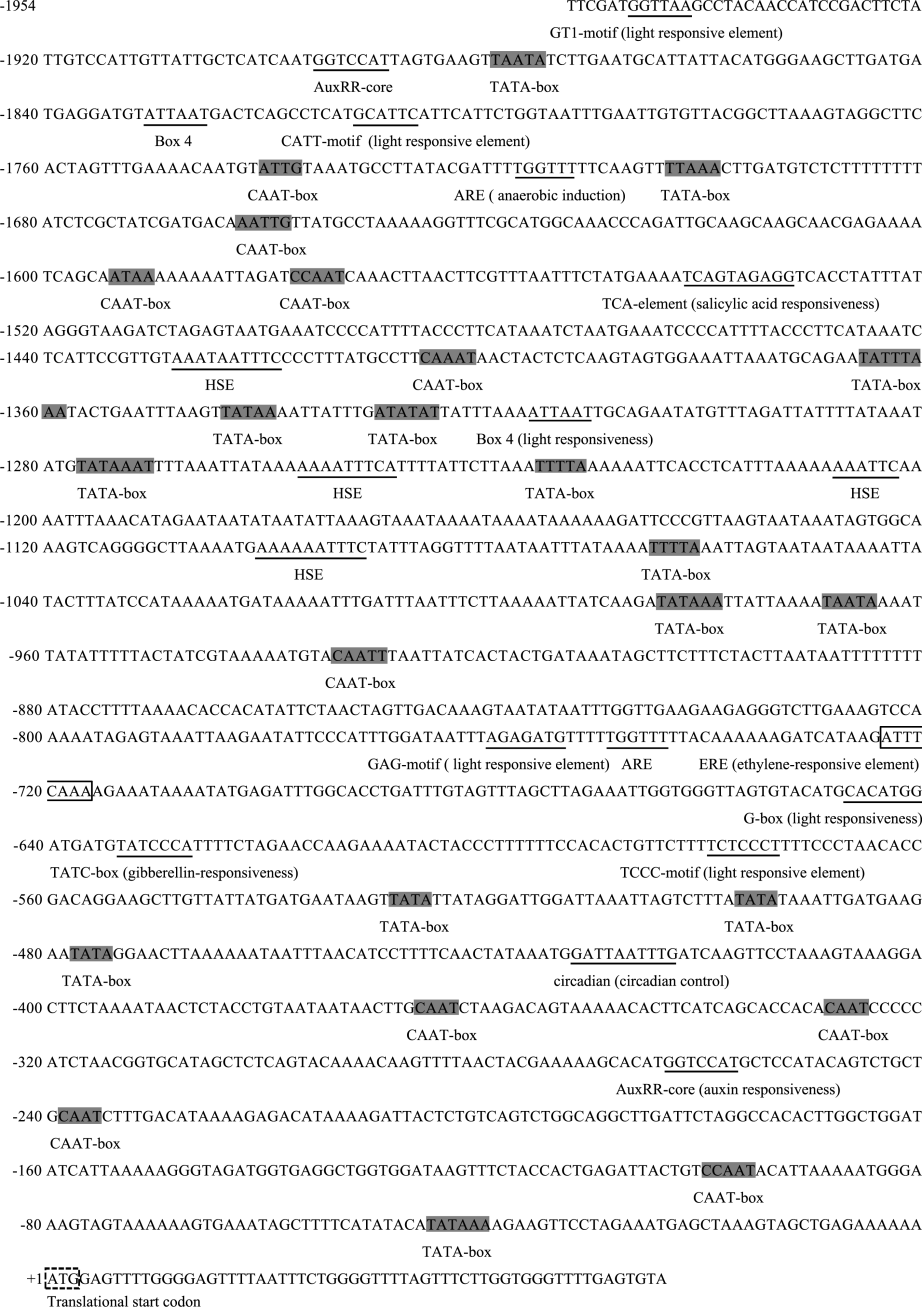
Analysis of *cis*-elements of *GhGLIP* promoter sequence. The translational start codon is indicated by a dashed line box. The TATA-box and CAAT-box are marked by a gray highlight. The ethylene-responsive element is indicated with a solid line box, and other responsive elements are indicated with an underline.

**Fig 9:**
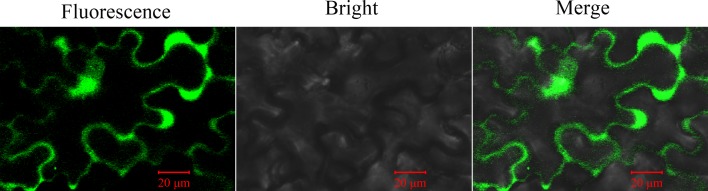
Activity of *GhGLIP* promoter driving GFP expression. The leaves of *pGhGLIP*::*GFP-GUS* transgenic tobacco plants were analyzed for *GhGLIP* promoter activity via GFP fluorescence. Bright field and merged images are also shown. GFP signal was detected by confocal microscopy.

**Fig 10:**
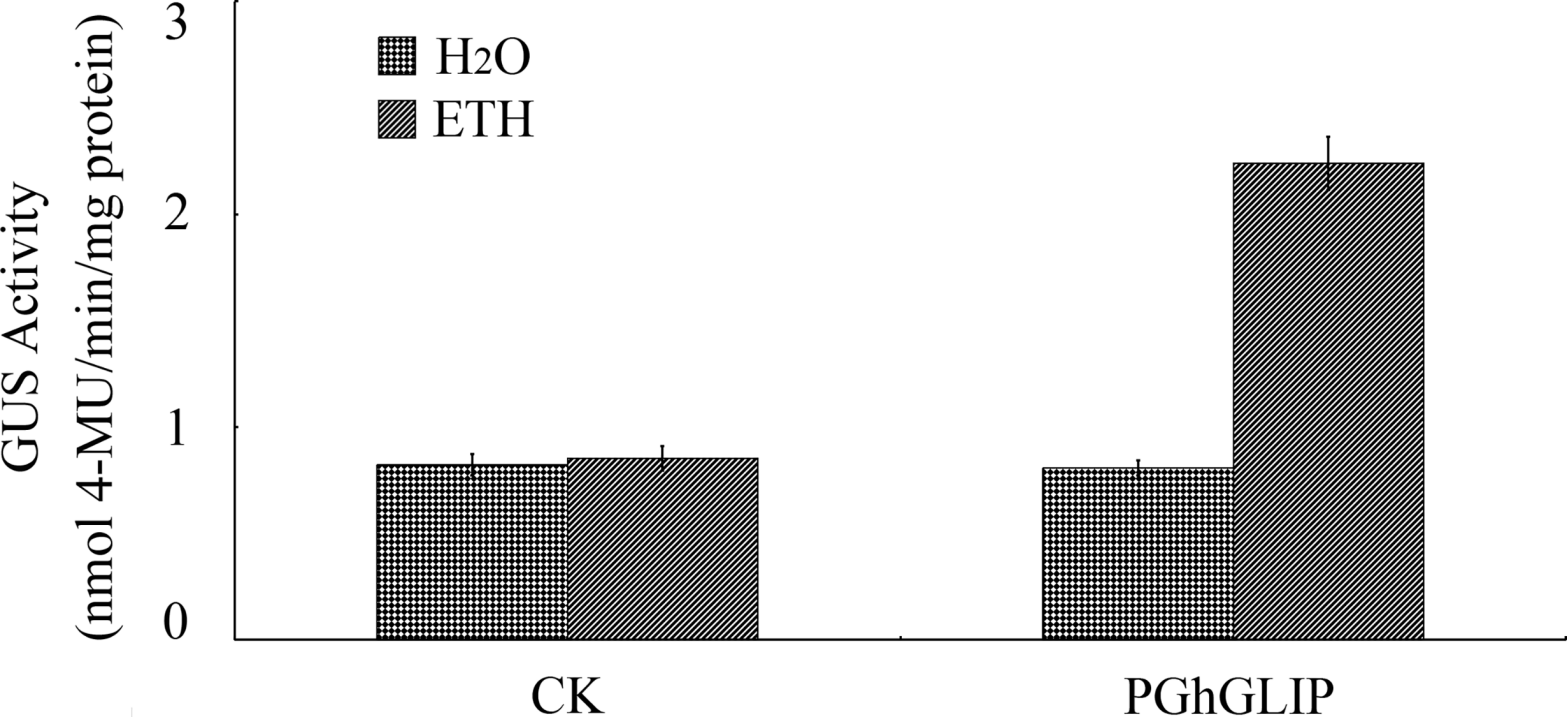
Activity of *GhGLIP* promoter under ethylene treatment. The leaves of *pGhGLIP*::*GFP-GUS* transgenic tobacco plants were treated with ethylene after which GUS activity was measured.

In the light of the crucial contributions of the plant hormone ethylene to cotton ovule and fiber development [10], and to regulating *GLIP* expression [21], *GhGLIP* expression under ethylene treatment was measured by qRT-PCR to determine the regulatory relationship between *GhGLIP* and ethylene. RNA samples, extracted from different treated ovule and fiber tissues through *in vitro* ovule culture, were used as templates for qRT-PCR analysis. The results showed that *GhGLIP* expression is significantly induced after 6 h treatment of ethylene (Fig 7). In addition, a 1,954-bp promoter sequence upstream of the coding region was obtained with reference to the *GhGLIP* cDNA sequence. The *GhGLIP* promoter (*pGhGLIP*) contained many putative plant *cis*-elements (listed in S3 Table), including TATA-boxes, CAAT-boxes, light responsive elements, and heat stress responsive elements (HSE), etc. Interestingly, some hormone-related elements were also explored, including the responsive elements of ethylene (ERE: ATTTCAAA), auxin (AuxRR-core: GGTCCA) and gibberellin (TATC-box: TATCCCA) (Fig 8). To evaluate the promoter activity, *pGhGLIP*::*GFP-GUS* was constructed and delivered into tobacco by Agrobacterium-mediated transient transformation. The green fluorescence of transformed tobacco leaves using the transient transformation method was detected using a laser confocal microscope (Fig 9), indicating that *pGhGLIP* shows high activity by activating the reporter gene *GFP* and that the *GhGLP* promoter is a complete functional sequence effectively driving gene expression. Moreover, the transgenic tobacco leaves transformed with *pGhGLIP*::*GFP-GUS* were treated with ETH to investigate the relationship between *pGhGLIP* expression and ethylene. The GUS enzyme activity showed a prompt increase in transgenic tobacco leaves after ETH treatment (Fig 10). These data suggest that *GhGLIP* expression is under the control of ethylene and may participate in ethylene-mediated signaling pathways.

## Conclusion and discussion

It has been validated that GDSL lipase affects plant leaf expansion, hypocotyl elongation and seed growth. Mostly, the *GLIP* gene has been studied in model plants such as Arabidopsis and rice, and in oil crops like *Brassica napus* and *Castor bean* [18–20, 21, 36]. We obtained the GDSL lipase gene *GhGLIP* from developing ovules and fibers of upland cotton. The mRNA expression and protein enzyme activity of *GhGLIP* were tightly related to ovule growth and fiber elongation (Fig 3). Cotton seeds are major resources of edible oil and high-quality protein, and contribute the protein requirements for half a billion people per year. Suppression of a cotton cytokinin dehydrogenase gene (*GhCKX*) resulted in enhanced seed size and increased yield of seed and fiber [37–38]. Suppression or overexpression of *GhSusA1* in transgenic cotton resulted in decrease of the fiber quality, boll size, and seed weight, or increase of fiber length and strength respectively [39]. As a part of the seed, the fiber is derived from the ovule epidermis and its growth is closely correlated with ovule development [1]. Cotton fiber yield promotion is positively linked with an increase in seed size [40–41], suggesting the important function of *GhGLIP* both in ovule and fiber development. It is an effective and necessary measurement to further investigate the *GhGLIP* important function in seed and fiber development through overexpression or suppression of *GhGLIP* in transgenic cotton.

GDSL lipases include conserved sequence features forming the typical motifs that are responsible for various functions within the classification of four common subfamily of clades I-V, with major roles in plant development and morphogenesis of clade I, stress response of clade II, biotic response of clade III and plant secondary metabolism of clade IV [18]. The function of clade IV GDSL lipase members, including Arabidopsis AtGELP 26 and 72 in seed fatty acid composition, AtGELP 33 (AtFXG1) in cell wall configuration, and *Brassica napu*s BnGLIP2 in seed germination, have been investigated [17, 42–43]. Cotton GhGLIP belonges to the clade IV GDSL lipase subfamily (Fig 2), suggesting its possible function in plant secondary metabolism. GhGLIP had four typical regions and one triplet catalytic center comprised of the amino acid residues Ser (S), Asp (D) and His (H) (Fig 1), providing the basis for its important function as a hydrolase, which is consistent with the report about GLIP structural characteristics in soybean, Arabidopsis and tobacco [17, 44–45]. The deduced GhGLIP protein maintained a leader peptide in the N terminus and two transmembrane regions (Fig 1) and localized in the plasma membrane, cytoplasm and nucleus (Fig 4), implying that GhGLIP may regulate intracellular gene expression and extracellular enzymatic reactions as a secretory protein. *Arab-1* was identified as member of the GDSL lipase family and fulfilled important roles in Arabidopsis development as an extracellular protein [46].

GDSL lipase has broad substrate specificity and regiospecificity that can hydrolyze glycerides, phospholipids and galactolipids and other ester compounds to generate kinds of fatty acids and sugars, which endows substances for plant growth and development. The GDSL lipase JNP1 isolated from *Jacaranda mimosifolia* enriched the content of free fatty acid in nectar [47]. Seed development was promoted significantly in transgenic plants overexpressing the *GLIP* gene. Ectopic expression of a *Brassica napus GLIP* gene *BnSCE3* in Arabidopsis resulted in enhanced weight and size of the transgenic seeds [48]. The transgenic line overexpressing the GDSL lipase gene in Arabidopsis exhibited better seedling growth [41]. We observed similar results with overexpressing *GhGLIP* transgenic Arabidopsis plants, especially promotion of plant seedling establishment (S1 Fig), seed length and fresh weight (Fig 5). Many predicted *GLIP* genes were involved seed development in Arabidopsis, rice, and *Brassica napus* [18–20]. These results demonstrate the critical function of *GhGLIP* in plant growth and seed development.

Seed development is determined by sugar level and protein storage of the cell. The Arabidopsis sugar metabolism gene *SuSy* altered dry weight accumulation, sugar and protein content of the seeds [35]. Overexpression of a potato *SuSy* gene increased the sugar amount, promoting cotton fiber elongation and seed development, including enriched seed fresh weight and enhanced seed set [3]. Cotton seed and fiber development was inhibited through repressing the expression of *SuSy* and was accompanied by diminished sugar content [4–5]. In transgenic Arabidopsis ectopically overexpressing cotton *GhGLIP*, a significant accumulation of sugar and protein was discovered in seeds, coupled with increased lipase activity (Fig 5).

Plant hormones such as auxin, gibberellic acid (GA) and ethylene, have been displayed that play important role in cotton fiber development. Supplement of indole-3-acetic acid (IAA) and GA was effective to promote fiber production *in vitro* [49]. Overexpression of the indole-3-acetic acid (IAA) biosynthetic gene *iaaM* improved the fiber fineness and increased the lint yield more than 15% in transgenic cotton [50]. In transgenic cotton overexpressing *GhGA20ox1*, both increase of endogenous GA levels and improvement of fiber quality and quantity were demonstrated [51].The expression of GDSL lipase is closely correlated with ethylene signaling. Ethylene induced the expression of Arabidopsis *AtGLIP1* [21, 25]. It has been indicated that ethylene is a crucial positive factor during fiber development and can significantly promote fiber quality through stimulating fiber cell elongation both *in vitro* and *in vivo* [10]. In cultured cotton ovules after treatment of ethylene, the genes *SuSy* and expansins (*EXP*), which are known to have important roles in cell wall biosynthesis or loosening and cell enlargement, were promoted significantly [10]. Expression of *GhGLIP* was induced after ethylene stimulation in cultured cotton ovules *in vitro* (Fig 7). The *GhGLIP* promoter was a functional sequence activating *GFP* expression and contained one ethylene responsive element, with induced expression under ETH stimulation (Fig 10), implying a potential possibility that *GhGLIP* may respond to ethylene through its functional promoter region. In conclusion, these results suggest that cotton *GhGLIP* may be involved in ovule and fiber development and promote seed growth.

## Supporting information

S1 Fig Representative phenotypes of transgenic Arabidopsis plants overexpressing *GhGLIP*. (TIF)

S2 Fig The expression level of *GhGLIP* in the transgenic Arabidopsis lines. (TIF)

S1 Table Primers used in this study. (DOCX)

S2 Table GenBank accession numbers of selected plant GDSL lipases used in this study. (DOC)

S3 Table List of *cis*-elements in promoter region of *GhGLIP*. (DOC)
