# Supplementary figures and images for: A Gossypium hirsutum GDSL lipase/hydrolase gene (GhGLIP) appears to be involved in promoting seed growth in Arabidopsis
Source: PLoS One. 2018 Apr 5;13(4):e0195556. doi: 10.1371/journal.pone.0195556 (PMC5886685; doi:10.1371/journal.pone.0195556)

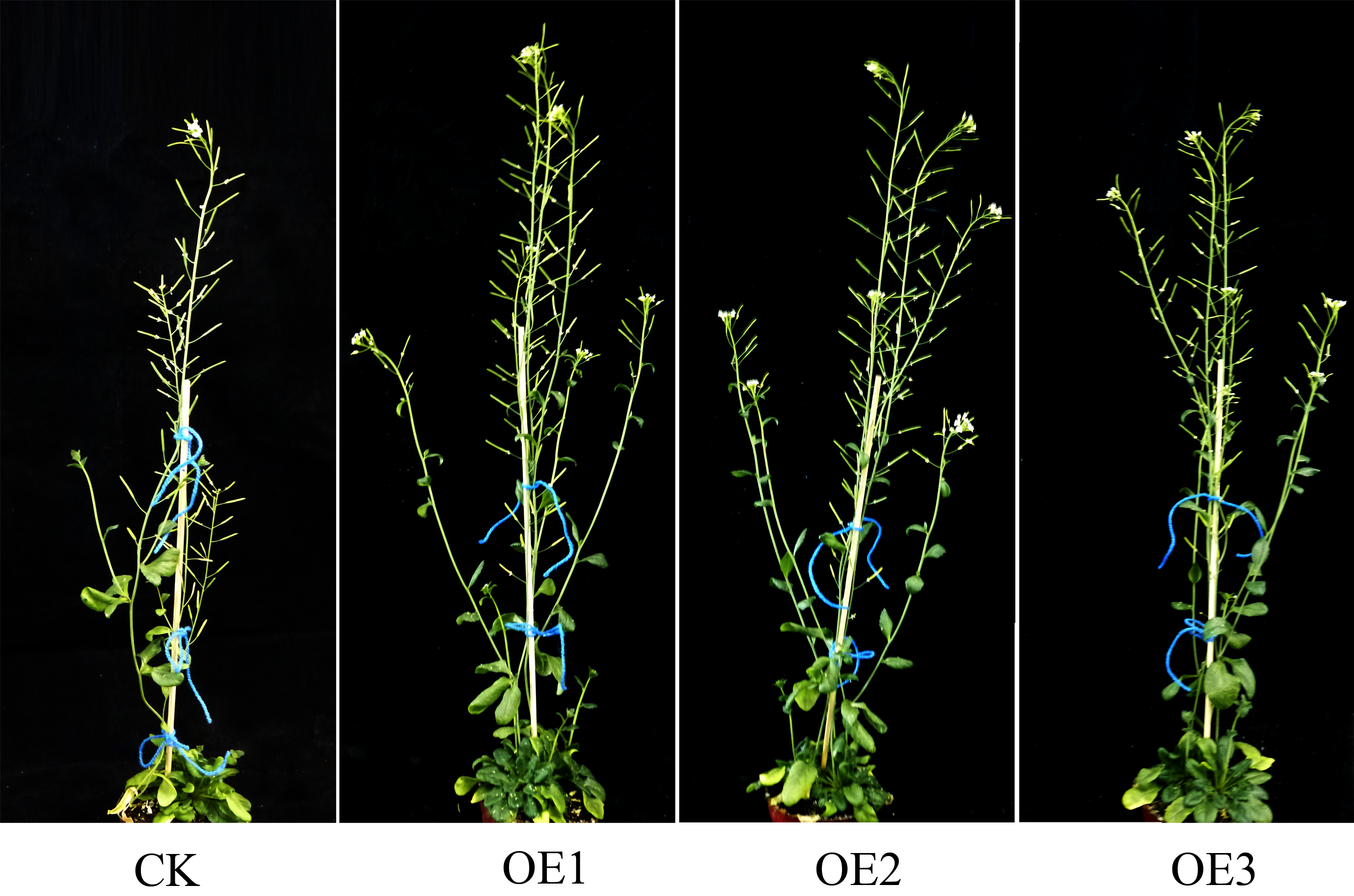

Supplement: S1 Fig — (TIF) [file pone.0195556.s001.tif]

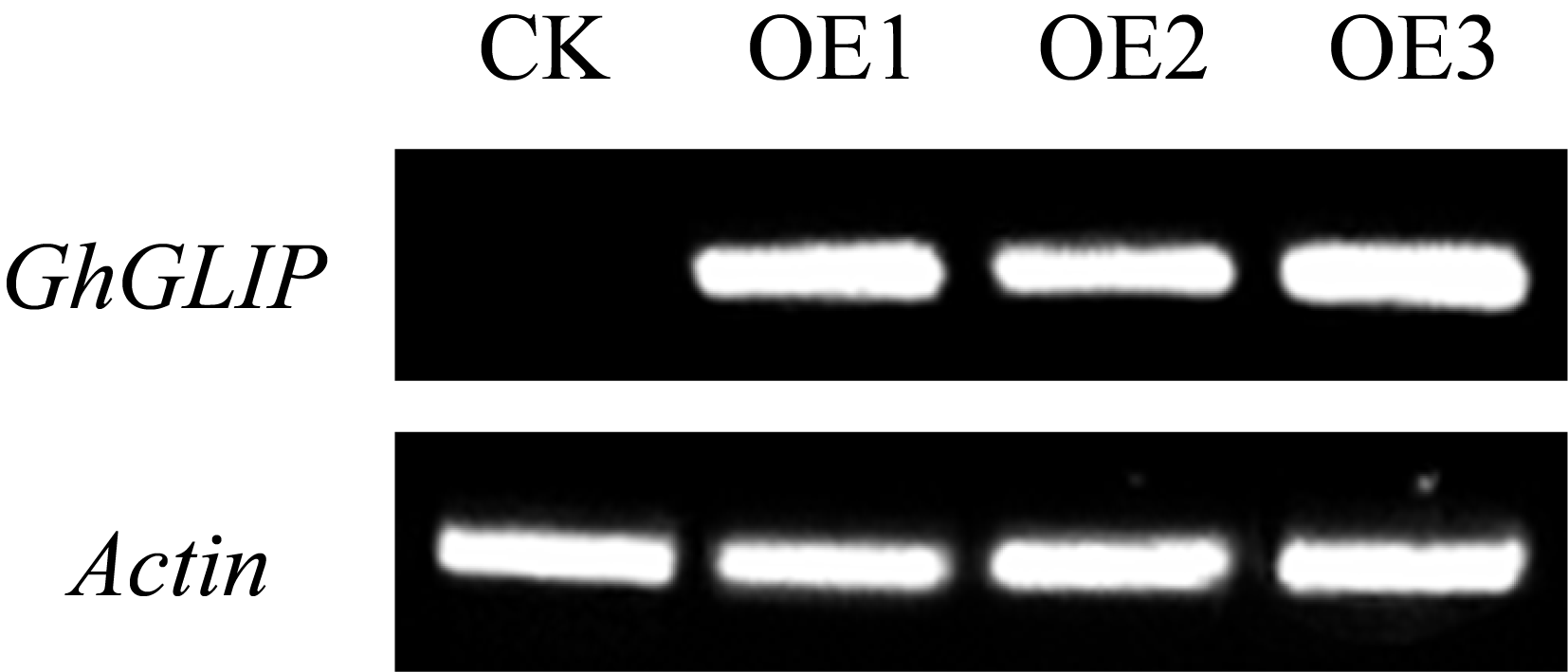

Supplement: S2 Fig — (TIF) [file pone.0195556.s002.tif]
