# Supplementary material for: A Gossypium hirsutum GDSL lipase/hydrolase gene (GhGLIP) appears to be involved in promoting seed growth in Arabidopsis
Source: PLoS One. 2018 Apr 5;13(4):e0195556. doi: 10.1371/journal.pone.0195556 (PMC5886685; doi:10.1371/journal.pone.0195556)
Supplement: S1 Table — (DOCX) [file pone.0195556.s003.docx]

S1 Table. Primers used in this study

| Primer name | Primer sequence (from 5’ to 3’) | Primer purpose |
| --- | --- | --- |
| *OCG-sense* | CGGGGTACCATGGAGTTTTGGGGAGTTTT | ORF cloning of *GhGLIP* cDNA (Underlines represent the restriction endonuclease sites of *Kpn* I in sense and *Xba* I in antisense ) |
| *OCG-antisense* | TGCTCTAGACTGCTTATGACATGCTTGAGTA |  |
| *VCG-sense* | CGGGGTACCATGGAGTTTTGGGGAGTTTT | Overexpression vector construction of *35S::GhGLIP-GFP*  (Underlines represent the restriction endonuclease sites of *Kpn* I in sense and *Xba* I in antisense ) |
| *VCG-antisense* | GCTCTAGACTGCTTATGACATGCTTGAGTA |  |
| *VCGP-sense* | CGGGATCCTTCGATGGTTAAGCCTACAACCATC | Vector construction of *pGhGLIP::GFP-GUS*  (Underlines represent the restriction endonuclease sites of *Bam*H I in sense and *Nco* I in antisense ) |
| *VCGP-antisense* | CATGCCATGGCCACCAAGAAACTAAAACCCCAGA |  |
| *RTG-sense* | GGTCGCCTCATCATTGACT | RT- and qRT-PCR detection of *GhGLIP* |
| *RTG-antisense* | GTGTATCCAGAAACTCCTCCC |  |
| *UBQ-sense* | AGAGGTCGAGTCTTCGGACA | qRT-PCR detection of *GhUBQ7* as internal control |
| *UBQ-antisense* | GCTTGATCTTGGGCTTG |  |
| *Actin- sense* | TGTGAGGGATGGAACAAATG | RT-PCR detection of *AtActin* as internal control |
| *Actin- antisense* | AACGCAGTTGCAAATAAAGGA |  |
