# Supplementary material for: A Gossypium hirsutum GDSL lipase/hydrolase gene (GhGLIP) appears to be involved in promoting seed growth in Arabidopsis
Source: PLoS One. 2018 Apr 5;13(4):e0195556. doi: 10.1371/journal.pone.0195556 (PMC5886685; doi:10.1371/journal.pone.0195556)
Supplement: S2 Table — (DOC) [file pone.0195556.s004.doc]

S2 Table GenBank accession numbers of selected plant GDSL lipases used in this study

| Protein name | Protein GenBank accession number | Protein purpose |
| --- | --- | --- |
| AtGLIP | *Arabidopsis thaliana* NP_196949.1 | Sequence alignment and conserved domain analysis |
| GmGLIP | *Glycine max* XP_003547701.1 |  |
| CmGLIP | *Cucumis melo* XP_008464936.1 |  |
| VvGLIP | *Vitis vinifera* XP_002271320.1 |  |
| CaGL1 | *Capsicum annuum* AAZ23955.1 | Phylogenetic tree construction |
| CaGLIP1 | *Capsicum annuum* AAX20033.1 |  |
| JNP1 | *Jacaranda mimosifolia* ABY59947.1 |  |
| BrSIL1 | *Brassica rapa subsp. Pekinensis* AAM47031.1 |  |
| Hev b 13 | *Hevea brasiliensis* Q7Y1X1.1 |  |
| LAE | *Digitalis lanata* CAA09694.1 |  |
| MaAChE | *Macroptilium atropurpureum* BAG09557.1 |  |
| ZmAChE | *Zea mays* BAD89850.1 |  |
| AAE | *Rauvolfia serpentine* AAW88320.1 |  |
| BnSCE3/BnLIP2 | *Brassica napus* AAX59709.1 |  |
| AmGDSH1 | *Alopecurus myosuroides* CAG27610.1 |  |
| Salicornia AChE | *Salicornia europaea* BAI23204.1 |  |
| AtGELP1 | *Arabidopsis thaliana* NP_563774.1 |  |
| AtGELP2 | *Arabidopsis thaliana* NP_172410.1 |  |
| AtGELP3 | *Arabidopsis thaliana* NP_564104.1 |  |
| AtGELP4 | *Arabidopsis thaliana* NP_173441.2 |  |
| AtGELP5 | *Arabidopsis thaliana* NP_173764.1 |  |
| AtGELP6 | *Arabidopsis thaliana* NP_174179.3 |  |
| AtGELP7 | *Arabidopsis thaliana* NP_174180.1 |  |
| AtGELP19 | *Arabidopsis thaliana* NP_175795.2 |  |
| AtGELP20 | *Arabidopsis thaliana* NP_175797.2 |  |
| AtGELP21 | *Arabidopsis thaliana* NP_175801.1 |  |
| AtGELP39 | *Arabidopsis thaliana* NP_565120.1 |  |
| AtGELP40 | *Arabidopsis thaliana* NP_565121.1 |  |
| AtGELP41 | *Arabidopsis thaliana* NP_177718.1 |  |
| AtGELP42 | *Arabidopsis thaliana* NP_177719.1 |  |
| AtGELP43 | *Arabidopsis thaliana* NP_565122.1 |  |
| AtGELP44 | *Arabidopsis thaliana* NP_177721.1 |  |
| AtGELP54 | *Arabidopsis thaliana* NP_180581.1 |  |
| AtGELP56 | *Arabidopsis thaliana* NP_180712.1 |  |
| AtGELP59 | *Arabidopsis thaliana* NP_181827.2 |  |
| AtGELP60 | *Arabidopsis thaliana* NP_187079.1 |  |
| AtGELP63 | *Arabidopsis thaliana* NP_188037.1 |  |
| AtGELP65 | *Arabidopsis thaliana* NP_188039.1 |  |
| AtGELP82 | *Arabidopsis thaliana* NP_194607.1 |  |
| AtGELP83 | *Arabidopsis thaliana* NP_194743.1 |  |
| AtGELP92 | *Arabidopsis thaliana* NP_568318.1 |  |
| AtGELP95 | *Arabidopsis thaliana* NP_198322.1 |  |
| AtGELP97 | *Arabidopsis thaliana* NP_198915.1 |  |
