# Supplementary material for: A Gossypium hirsutum GDSL lipase/hydrolase gene (GhGLIP) appears to be involved in promoting seed growth in Arabidopsis
Source: PLoS One. 2018 Apr 5;13(4):e0195556. doi: 10.1371/journal.pone.0195556 (PMC5886685; doi:10.1371/journal.pone.0195556)
Supplement: S3 Table — (DOC) [file pone.0195556.s005.doc]

S3 Table. List of *cis*-elements in promoter region of *GhGLIP*

| Element Name | Position  (+, plus strand; -, minus strand) | Element Sequence | Element Function |
| --- | --- | --- | --- |
| GT1-motif | -1942(+) | GGTTAA | light responsive element |
| AuxRR-core  CATT-motif | -1888(+), -257(+)  -1870(-), -1872(+), -743(+) | GGTCCAT  GCATTC | auxin responsiveness  light responsive element |
| O_2_-site | -1845(+) | GATGATGTGG | zein metabolism regulation |
| Box 4  Skn-1_motif  GCN4_motif | -1835(+), -1310(+), -389(-)  -1832(-), -1723(-)  -1879(-) | ATTAAT  GTCAT  TGAGTCA | light responsiveness  endosperm expression  endosperm expression |
| Box I  Box III  I-box  ARE  AT-rich element | -1808(-)  -1791(-)  -1783(-)  -1772(+), -1071(+)  -1546(-) | TTTCAAA  CATTTACACT  GTATAAGGCC  TGGTTT  ATAGAAATCAA | light responsive element  protein binding site  light responsive element  anaerobic induction  AT-rich DNA binding protein (ATBP-1) |
| TCA-element  TCT-motif  CCAAT-box | -1532(+)  -1511(-)  -1429(-) | TCAGTAGAGG  TCTTAC  CAACGG | salicylic acid responsiveness  light responsive element  MYBHv1 binding site |
| HSE | -1418(+)  -1248(+)  -1202(+)  -1091(+) | AAATAATTTC  AAAATTTCA  AAATTC  AAAAAATTTC | heat stress responsiveness |
| AT1-motif  chs-CMA1a  CAT-box  MRE | -1310(-)  -1132(-)  -1121(-)  -1081(-) | ATTAATTTTACA  TTACTTAA  GCCACT  AACCTAA | light responsive module  light responsive element  meristem expression  MYB binding site involved in light responsiveness |
| GAG-motif | -753(+) | AGAGATG | light responsive element |
| ERE  Box III | -716(+)  --647(-) | ATTTCAAA  CATTTACACT | ethylene-responsive element  protein binding site |
| G-box | -641(+), -263(+) | CACATGG | light responsiveness |
| TATC-box | -627(+) | TATCCCA | gibberellin-responsiveness |
| TCCC-motif  ATCT-motif  TC-rich repeats | -537(+)  -469(-), -474(-)  -439(-) | TCTCCCT  AATCTAATCC  ATTTTCTTCA | light responsive element  light responsiveness  defense and stress responsiveness |
| Circadian | -739(-)  -423(-) | CAAAAACATC  CAAATTAATC | circadian control |
| ATC-motif  AE-box | -205(-), -103(-)  -128(-) | AGTAATCT  AGAAACTT | light responsiveness  light response |
